# Identification of Matriglycan by Dual Exoglycosidase Digestion of α-Dystroglycan

**DOI:** 10.21769/BioProtoc.4827

**Published:** 2023-09-20

**Authors:** Ishita Chandel, Kevin P. Campbell

**Affiliations:** Howard Hughes Medical Institute, Senator Paul D. Wellstone Muscular Dystrophy Specialized Research Center, Department of Molecular Physiology and Biophysics and Department of Neurology, Roy J. and Lucille A. Carver College of Medicine, The University of Iowa, Iowa City, IA, USA

**Keywords:** Matriglycan, Dystroglycan, Exoglycosidases, β-Glucuronidase, α-Xylosidase, Thermophiles, Glycosylation, Digestion

## Abstract

_Matriglycan is a linear polysaccharide of alternating xylose and glucuronic acid units [-Xyl-α1,3-GlcA-β1,3]_n that is uniquely synthesized on α-dystroglycan (α-DG) and is essential for neuromuscular function and brain development. It binds several extracellular matrix proteins that contain laminin-globular domains and is a receptor for Old World arenaviruses such as Lassa Fever virus. Monoclonal antibodies such as IIH6 are commonly used to detect matriglycan on α-DG. However, endogenous expression levels are not sufficient to detect and analyze matriglycan by mass spectrometry approaches. Thus, there is a growing need to independently confirm the presence of matriglycan on α-DG and possibly other proteins. We used an enzymatic approach to detect matriglycan, which involved digesting it with two thermophilic exoglycosidases: β-Glucuronidase from Thermotoga maritima and α-xylosidase from Sulfolobus solfataricus. This allowed us to identify and categorize matriglycan on α-DG by studying post-digestion changes in the molecular weight of α-DG using SDS-PAGE followed by western blotting with anti-matriglycan antibodies, anti-core α-DG antibodies, and/or laminin binding assay. In some tissues, matriglycan is capped by a sulfate group, which renders it resistant to digestion by these dual exoglycosidases. Thus, this method can be used to determine the capping status of matriglycan. To date, matriglycan has only been identified on vertebrate α-DG. We anticipate that this method will facilitate the discovery of matriglycan on α-DG in other species and possibly on other proteins.

Key features

• Analysis of endogenous matriglycan on dystroglycan from any animal tissue.

• Matriglycan is digested using thermophilic enzymes, which require optimum thermophilic conditions.

• Western blotting is used to assay the success and extent of digestion.

• Freshly purified enzymes work best to digest matriglycan.

**Graphical overview**


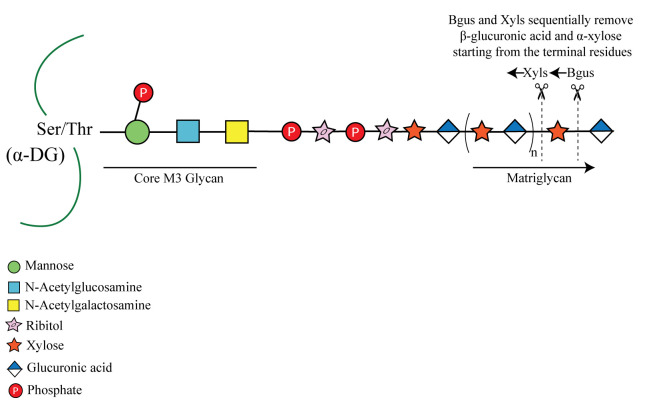


α-Dystroglycan (α-DG) from muscle is shown here modified by a phosphorylated core M3 glycan, which extends further and terminates in a repeating disaccharide of xylose (Xyl) and glucuronic acid (GlcA) called matriglycan. β-glucuronidase (Bgus) and α-xylosidase (Xyls) hydrolyze the β-1,3-linked GlcA and α-1,3 linked-Xyl, starting from the terminal residues.

## Background

α-Dystroglycan (α-DG) is an extensively glycosylated and widely expressed transmembrane protein that functions as an extracellular matrix receptor. It is modified with several types of O- and N- glycans. The glycans on α-DG enable it to interact with the extracellular molecules and maintain cell stability and integrity. The most widely studied post-translational modification of α-DG is the addition of the *O*-mannosylated glycan called core M3. This glycan is defined as a phosphorylated *O*-mannosyl trisaccharide [GalNAcβ1-3GlcNAcβ1-4(phosphate-6)Man-*O*-Ser] comprised of a mannose that is extended by the sequential addition of an N-acetylglucosamine and an N-acetyl galactosamine (Yoshida-Moriguchi and Campbell, 2015). The C6 hydroxyl group of the mannose of core M3 is also phosphorylated by a kinase (Yoshida-Moriguchi et al., 2013). Core M3 is further elongated, from the N-acetylgalactosamine, by a glycan modification that terminates in the repeating disaccharide of xylose (Xyl) and glucuronic acid [-Xyl-α1,3-GlcA-β1,3-]_n_ called matriglycan. Notably, over 18 genes are involved in generating the final core M3 structure (Walimbe et al., 2020). Matriglycan acts as a scaffold for laminin-G domain containing proteins (e.g., laminin, agrin, perlecan, and neurexin) in the extracellular matrix. Loss-of-function mutations in any of the 18 genes involved in matriglycan synthesis lead to different types of muscular dystrophies, including severe forms such as Walker-Warburg syndrome.

Owing to the complexity of the final core M3 structure terminating in matriglycan and its low expression levels in cells, it has never been analyzed using mass spectrometry. Therefore, to study and to confirm the presence of matriglycan on dystroglycan in various animal tissues, mutants, and patient samples, we use an enzymatic approach. We identified two exoglycosidases that together can hydrolyze matriglycan in native tissues: β-glucuronidase (Bgus) from *Thermotoga maritima* (Salleh et al., 2006) and α-xylosidase (Xyls) from *Sulfolobus solfataricus* (Moracci et al., 2000). In conjunction with mass spectrometry and chemically synthesized matriglycan, we previously demonstrated that these enzymes sequentially remove sugars from the non-reducing end and that Bgus and Xyls specifically cleave the β1,3-linked GlcA and α1,3-linked Xyl, respectively (Briggs et al., 2016). Given that neither enzyme has detectable endoglycosidase activity (Briggs et al., 2016), they enable us to probe the matriglycosylation status of native α-DG. Matriglycan from brain was found to be capped by a 3-O-sulfation of its terminal GlcA, a reaction catalyzed by HNK-1 sulfotransferase. The sulfate cap prevents further elongation of matriglycan and also makes it resistant to digestion by the dual exoglycosidases. It has been previously demonstrated that the matriglycan on α-DG isolated from brain is capped by sulfation and becomes susceptible to digestion by the exoglycosidases after digesting it with a sulfatase (Sheikh et al., 2020). Therefore, this method enables us to probe the tissue-dependent matriglycosylation status of α-DG. To date, matriglycan has been found only on α-DG, although a report suggests it could be present on glypican-4 (Inamori et al., 2016). This digestion protocol is expected to be useful in confirming the presence of matriglycan on other proteins such as glypican and may also aid in discovering new proteins modified with matriglycan. Also, matriglycan has been found only in vertebrate α-DG, e.g., in human, mouse, and zebrafish (Praissman et al., 2016; Liu et al., 2020), and this protocol can be used to determine whether matriglycan exists in other species. Such analyses have the potential to broaden the use of other species as a model organism for the study of muscular dystrophies arising from changes in matriglycosylation of α-DG. Although matriglycan can be detected using antibodies, those currently available sometimes detect nonspecific bands; therefore, confirming the presence of matriglycan independently will be important, especially when working with new proteins or organisms. In summary, the technique described here is expected to be useful for definitively detecting matriglycan (in contrast to antibodies) on new proteins and in new organisms.

One limitation of using thermophilic enzymes for digestion is that they require high temperatures and acidic pH to function, which could be detrimental to some substrate proteins. We have found α-DG to remain remarkably stable at high temperatures and acidic pH, making the use of the dual thermophilic exoglycosidases feasible (Briggs et al., 2016).

## Materials and reagents

**Biological materials**

1. BL21(DE3) chemically competent *E. coli* cells (Invitrogen, One Shot, C600003)
2. C57BL/6J mice (The Jackson Laboratory, 000664)

**Reagents**

1. BGUS_pET-28a(+) (GenScript, SC1691, custom-made)
2. XylS_pET-28a(+) (GenScript, SC1691, custom-made)
3. IPTG (Isopropylthio-β-galactoside) (IBI Scientific, catalog number: IB02105)
4. Kanamycin (IBI Scientific, catalog number: IB02120)
5. TALON superflow metal affinity resin (Takara Bio, catalog number: 635506)
6. Econo-Column chromatography columns, 1.5 cm × 10 cm, glass (Bio-Rad, catalog number: 7371512)
7. Nuclease, Pierce Universal nuclease (Thermo Scientific, catalog number: 88701)
8. 30 KD cutoff concentrators, Amicon Ultra centrifugal filters (Sigma-Aldrich, catalog number: UFC803024)
9. Coomassie stain, G-250 stain (Bio-Rad, catalog number: 1610786)
10. 2-mercaptoethanol (VWR, catalog number: 97064-880)
11. Wheat germ agglutinin (WGA), agarose bound (Vector Laboratories, catalog number: AL-1023-10)
12. Core DG antibody (Bio-Techne, R&D systems, catalog number: AF6868)
13. Anti-matriglycan antibody (DSHB, catalog number: IIH6 C4)
14. SOC medium (Invitrogen, One Shot, catalog number: C600003)
15. Acrylamide, 30% Acrylamide/Bis solution, 37.5:1 (Bio-Rad, catalog number: 1610158)
16. Ammonium persulfate (APS) (Sigma-Aldrich, catalog number: 248614-500G)
17. TEMED (RPI, catalog number: T18000-0.05)
18. N-Acetylglucosamine (Sigma-Aldrich, catalog number: A8625)
19. Bacto-Agar (BD Difco, catalog number: 214010)
20. Bacto-Tryptone (BD Difco, catalog number: 211705)
21. NaCl (IBI Scientific, catalog number: IB07071)
22. Yeast extract (BD Difco, catalog number: 212750)
23. Tris (Roche, catalog number: 11814273001)
24. Triton X-100 (Fisher Chemicals, catalog number: BP151)
25. Pepstatin (EMD-Millipore, catalog number: 516481)
26. PMSF (Phenylmethylsulfonyl fluoride) (Sigma-Aldrich, catalog number: P7626)
27. Benzamidine hydrochloride hydrate (MP Biomedicals, catalog number: 195068)
28. Calpeptin (EMD-Millipore, catalog number: 03-34-0051)
29. Calpain inhibitor (Sigma-Aldrich, catalog number: A6185)
30. Leupeptin (Sigma-Aldrich, catalog number: EI8)
31. Aprotinin (Sigma-Aldrich, catalog number: A3886)
32. Glycine (Bio-Rad, catalog number: 1610718)
33. Methanol (Sigma-Aldrich, catalog number: 179337)
34. KCl (potassium chloride) (Fisher Chemicals, catalog number: P217)
35. Na_2_HPO_4_ (sodium dibasic monophosphate) (Fisher Chemicals, catalog number: S374)
36. KH_2_PO_4_ (potassium dihydrogen phosphate) (Sigma-Aldrich, catalog number: P5655)
37. CaCl_2_·2H_2_O (calcium chloride) (Sigma-Aldrich, catalog number: C8106)
38. MgCl_2_·6H_2_O (magnesium chloride hexahydrate) (Fisher Chemicals, catalog number: M33)
39. Sodium acetate (Fisher Chemicals, catalog number: BP334)
40. SDS (sodium dodecyl sulfate) (SERVA, catalog number: 20765)
41. EDTA (ethylenediaminetetraacetic acid) (PRIMA, catalog number: KCE14000)
42. Filtration system PES 0.45 µm (Cole-Parmer, catalog number: UX-07630-08)

**Solutions**

1. Luria-Bertani (LB) medium (see Recipes)
2. Luria-Bertani (LB) agar plates-Kanamycin (see Recipes)
3. Lysis buffer (see Recipes)
4. Wash buffer 1 (see Recipes)
5. High salt wash buffer (see Recipes)
6. Elution buffer (see Recipes)
7. Solubilization buffer (see Recipes)
8. WGA wash buffer (see Recipes)
9. WGA elution buffer (see Recipes)
10. 10× Phosphate buffered saline (PBS) pH 7.4 (see Recipes)
11. Sodium acetate buffer (see Recipes)
12. Two (3%–15%) SDS-PAGE separating gradient gels (see Recipes)
13. Two stacking gels (see Recipes)
14. Separating gel buffer (see Recipes)
15. Stacking gel buffer (see Recipes)
16. 10× Western blot transfer buffer (see Recipes)
17. 1× Western transfer tank buffer (see Recipes)

**Recipes**

1. **Luria-Bertani (LB) medium**
  **Table:** 

Reagent	Final concentration	Quantity
Bacto-Tryptone	1%	10 g
Yeast extract	0.5%	5 g
NaCl	1%	10 g
H_2_O	n/a	1,000 mL
Total	n/a	1,000 mL
Autoclave for 30 min Store at room temperature
2. **Luria-Bertani (LB) agar plates-Kanamycin**
  **Table:** 

Reagent	Final concentration	Quantity
Bacto-Tryptone	1%	10 g
Yeast extract	0.5%	5 g
NaCl	1%	10 g
Agar	2%	20 g
Kanamycin	50 μg/mL	50 μL (from 50 mg/mL stock)
H_2_O	n/a	1,000 mL
Total	n/a	1,000 mL
Autoclave for 30 min Note: Add Kanamycin after media has cooled down to 65–70 °C. Store plates at 4 °C
3. **Lysis buffer**
  **Table:** 

Reagent	Final concentration	Quantity	Stock concentration
Tris-HCl	50 mM	2.5 mL	1 M
NaCl	100 mM	1.25 mL	4 M
Triton X-100	1%	2.5 mL	20%
Pepstatin A	0.6 μg/mL	0.03 mL	1 mg/mL
Aprotinin	0.5 μg/mL	5 μL	5 mg/mL
Leupeptin	0.5 μg/mL	5 μL	5 mg/mL
Calpain I inhibitor	5 μM	0.05 mL	5 mM
Calpeptin	5 μM	0.05 mL	5 mM
PMSF	0.1 mM	0.05 mL	0.1 M
Benzamidine	0.75 M	0.375 mL	0.1 M
Total	n/a	50 mL	
Always prepare fresh
4. **Wash buffer 1**
  **Table:** 

Reagent	Final concentration	Quantity	Stock concentration
Tris-HCl	50 mM	5 mL	1 M
NaCl	100 mM	2.5 mL	4 M
Triton X-100	0.1%	0.5 mL	20%
Pepstatin A	0.6 μg/mL	0.06 mL	1 mg/mL
Aprotinin	0.5 μg/mL	0.01 mL	5 mg/mL
Leupeptin	0.5 μg/mL	0.01 mL	5 mg/mL
Calpain I inhibitor	5 μM	0.1 mL	5 mM
Calpeptin	5 μM	0.1 mL	5 mM
PMSF	0.1 mM	0.1 mL	0.1 M
Benzamidine	0.75 mM	0.750 mL	0.1 M
Total	n/a	100 mL	
Always prepare fresh
5. **High salt wash buffer**
  **Table:** 

Reagent	Final concentration	Quantity	Stock concentration
Tris-HCl	50 mM	2.5 mL	1 M
NaCl	500 mM	6.25 mL	4 M
Triton X-100	0.1%	0.25 mL	20%
Pepstatin A	0.6 μg/mL	0.03 mL	1 mg/mL
Aprotinin	0.5 μg/mL	5 μL	5 mg/mL
Leupeptin	0.5 μg/mL	5 μL	5 mg/mL
Calpain I inhibitor	5 μM	0.05 mL	5 mM
Calpeptin	5 μM	0.05 mL	5 mM
PMSF	0.1 mM	0.05 mL	0.1 M
Benzamidine	0.75 mM	0.375 mL	0.1 M
Total	n/a	50 mL	
Always prepare fresh
6. **Elution buffer**
  **Table:** 

Reagent	Final concentration	Quantity	Stock concentration
Tris-HCl	50 mM	2.5 mL	1 M
NaCl	100 mM	1.25 mL	4 M
Triton X-100	0.1%	0.25 mL	20%
Imidazole	300 mM	15 mL	1 M
Pepstatin A	0.6 μg/mL	0.03 mL	1 mg/mL
Aprotinin	0.5 μg/mL	5 μL	5 mg/mL
Leupeptin	0.5 μg/mL	5 μL	5 mg/mL
Calpain I inhibitor	5 μM	0.05 mL	5 mM
Calpeptin	5 μM	0.05 mL	5 mM
PMSF	0.1 mM	0.05 mL	0.1 M
Benzamidine	0.75 mM	0.375 mL	0.1 M
Total	n/a	50 mL	
Always prepare fresh
7. **Solubilization buffer**
  **Table:** 

Reagent	Final concentration	Quantity	Stock Concentration
Tris-HCl	50 mM	2.5 mL	1 M
NaCl	100 mM	1.25 mL	4 M
Triton X-100	1%	2.5 mL	20%
EDTA	10 mM	1 mL	0.5 M
Pepstatin A	0.6 μg/mL	0.03 mL	1 mg/mL
Aprotinin	0.5 μg/mL	5 μL	5 mg/mL
Leupeptin	0.5 μg/mL	5 μL	5 mg/mL
Calpain I inhibitor	5 μM	0.05 mL	5 mM
Calpeptin	5 μM	0.05 mL	5 mM
PMSF	0.1 mM	0.05 mL	0.1 M
Benzamidine	0.75 mM	0.375 mL	0.1 M
Total	n/a	50 mL	
Always prepare fresh
8. **WGA wash buffer**
  **Table:** 

Reagent	Final concentration	Quantity	Stock concentration
Tris-HCl	50 mM	2.5 mL	1 M
NaCl	100 mM	1.25 mL	4 M
Triton X-100	0.1%	0.25 mL	20%
EDTA	10 mM	1 mL	0.5 M
Pepstatin A	0.6 μg/mL	0.03 mL	1 mg/mL
Aprotinin	0.5 μg/mL	5 μL	5 mg/mL
Leupeptin	0.5 μg/mL	5 μL	5 mg/mL
Calpain I inhibitor	5 μM	0.05 mL	5 mM
Calpeptin	5 μM	0.05 mL	5 mM
PMSF	0.1 mM	0.05 mL	0.1 M
Benzamidine	0.76 mM	0.376 mL	0.1 M
Total	n/a	50 mL	
Always prepare fresh
9. **WGA elution buffer**
  **Table:** 

Reagent	Final concentration	Quantity	Stock concentration
Tris-HCl	50 mM	2.5 mL	1 M
NaCl	100 mM	1.25 mL	4 M
Triton X-100	0.1%	0.25 mL	20%
EDTA	10 mM	1 mL	0.5 M
N-acetylglucosamine	0.3 M	3.31 grams	n/a
Pepstatin A	0.6 μg/mL	0.03 mL	1 mg/mL
Aprotinin	0.5 μg/mL	5 μL	5 mg/mL
Leupeptin	0.5 μg/mL	5 μL	5 mg/mL
Calpain I inhibitor	5 μM	0.05 mL	5 mM
Calpeptin	5 μM	0.05 mL	5 mM
PMSF	0.1 mM	0.05 mL	0.1 M
Benzamidine	0.77 mM	0.377 mL	0.1 M
Total	n/a	50 mL	
Always prepare fresh
10. **10× Phosphate buffered saline (PBS)**
  **Table:** 

Reagent	Final concentration	Quantity
NaCl	1.37 M	80 g
KCl	27 mM	2 g
Na_2_HPO_4_	100 mM	14.4 g
KH_2_PO_4_	18 mM	2.4 g
CaCl_2_·2H_2_O	10 mM	1.33 g
MgCl_2_·6H_2_O	5 mM	1.0 g
H_2_O	n/a	1,000 mL
Adjust pH to 7.4 with HCl		
Store at room temperature
11. **Sodium acetate buffer**
  **Table:** 

Reagent	Final concentration	Quantity
Sodium acetate	150 mM	10.2 g
Adjust pH to 5.5		
H_2_O	n/a	500 mL
Total Store at room temperature	n/a	500 mL
12. **Two (3%–15%) SDS-PAGE separating gradient gels**
  **Table:** 

Reagent	3%	15%
Water	19.3 mL	7.3 mL
Separating gel buffer	7.5 mL	7.5 mL
Acrylamide	3 mL	15 mL
TEMED	11 μL	11 μL
10% APS	180 μL	180 μL
Prepare fresh 10% APS
13. **Two stacking gels**
  **Table:** 

Reagent	Quantity
Water	13.3 mL
Stacking gel buffer	5 mL
Acrylamide	2.3 mL
TEMED	15 μL
10% APS	150 μL
Prepare fresh 10% APS
14. **Separating gel buffer**
  **Table:** 

Reagent	Final concentration	Quantity
Tris Adjust pH to 8.8 with HCL	1.5 M	90 g
SDS	0.4%	2 g
H_2_O	n/a	500 mL
Total	n/a	500 mL
Sterilize through 0.45 μm filter Store at room temperature
15. **Stacking gel buffer**
  **Table:** 

Reagent	Final concentration	Quantity
Tris Adjust pH to 6.8 with HCL	0.5 M	30 g
SDS	0.4%	2 g
H_2_O	n/a	500 mL
Total	n/a	500 mL
Sterilize through 0.45 μm filter Store at room temperature
16. **10× Western blot transfer buffer**
  **Table:** 

Reagent	Quantity
Glycine	288 g
Tris base	60.6 g
Water	2,000 mL
Store at room temperature
17. **1× Western transfer tank buffer**
  **Table:** 

Reagent	Quantity
10× transfer buffer	400 mL
100% methanol	800 mL
Water	2,800 mL
Total	4,000 mL

**Laboratory supplies**

1. Rattler plating beads, 5 mm (Zymo Research, catalog number: S1001)
2. Liquid scintillation vials (RPI, catalog number: 121043)
3. Glass homogenizer (Cole-Parmer, catalog number: EW-44468-18)
4. Plain plunger (Cole-Parmer, EW-44468-06)
5. PVDF membrane (Immobilon-FL, Millipore, catalog number: IPFL00010)
6. Blotting paper (Cytiva Whatman 3MM CHR, catalog number: 3030-917)
7. Petri dishes (Fisher Scientific, catalog number: FB0875713)
8. Scissors (FST, catalog number: 14090-11)
9. Forceps (FST, catalog number: 91150-20)
10. Mortar and pestle (Fisher Scientific, catalog number: S39831)

## Equipment

1. Thermomixer (Eppendorf, catalog number: 5382000023)
2. Thermo Top (Eppendorf, catalog number: 5308000003)
3. Polytron homogenizer (Fisher Scientific, Kinematica Polytron PT 2500 E homogenizer, catalog number: 08-451-320)
4. Generator/probe for Polytron homogenizer (Fisher Scientific, Kinematica, catalog number: 05-400-263)
5. Spectrophotometer (Eppendorf Bio photometer, model: 6131)
6. Water bath (Fisher Scientific, Isotemp model: 205)
7. Incubator (VWR, New Brunswick, Innova 42, catalog number: 75875-638)
8. Rotator (ATR Rotamix, model: RKVSD)
9. Motorized overhead stirrer (Fisher Scientific, DWK Life Sciences Wheaton, catalog number: 22-244382)
10. SDS-PAGE gradient gel system (Hoefer SE 600 model, 18 cm × 16 cm)
11. Western blot transfer system (Hoefer, TE 42 model)

## Procedure

- **Gene cloning**
  1. β-glucuronidase (Bgus) from *Thermotoga maritima* and α-xylosidase (Xyls) from *Sulfolobus solfataricus* were cloned into pET-28a(+) vector between NheI/XhoI sites in frame with the N-terminal 6x-His tag by GenScript (see supplemental file S1).
- **Cell transformation**
  1. Thaw BL21DE3 competent cells on ice (50 μL aliquot for each plasmid).
  2. Add 20 ng of each plasmid to the competent cells (mix by swirling the pipette tip three times).
  3. Incubate cells in ice for 30 min.
  4. Heat shock the cells at 42 °C for 30 s.
  5. Incubate on ice again for 5 min. **Critical:** This step is required to obtain high efficiency of transformation.
  6. Add 950 μL of SOC medium (at room temperature) and incubate at 37 °C for 60 min with shaking at 350 rpm in a thermomixer.
  7. For each sample, spread 50 μL from each tube onto LB agar plates with Kanamycin (use plating beads or spreader).
  8. Incubate the plates at 37 °C for 16 h.
- **Purification of Bgus and Xyls**
  1. Pick a single colony from each plate and inoculate 20 mL of LB medium (with Kanamycin, 50 μg/mL). Incubate at 37 °C for 16 h with shaking at 200 rpm.
  2. Use 10 mL of the overnight culture to inoculate 1 L of LB medium (with Kanamycin, 50 μg/mL). Incubate at 37 °C with shaking at 200 rpm. Keep checking the OD_600_.
  3. Once the OD_600_ reaches 0.6, add 1 mM IPTG (1.6 mL from a 600 mM stock solution) to the culture to induce gene expression and incubate at 16 °C for 16 h with shaking at 200 rpm.
  4. Centrifuge the cells at 5,000× *g* for 10 min at 4 °C.
  5. Store the cell pellets at -80 °C until ready for the purification step. **Pause point:** Cell pellets can be stored long term if necessary.
  6. Dissolve the cell pellets in lysis buffer (20 mL of buffer per liter of starting culture).
  7. Store the cells at -80 °C overnight to allow ice crystals to form; this step aids in cell lysis.
  8. Thaw the cells at room temperature for purification.
  9. Add nuclease at 6.25 kU (5 μL) concentration and sonicate cells for 5 s (power 4–5) four times with 10 s intervals at 4 °C.
  10. Centrifuge cells at 15,000× *g* for 20 min at 4 °C. Place supernatant into a new tube.
  11. Place the supernatant into a 75 °C water bath for 10 min. **Critical:** This step is essential to enrich the thermophilic enzymes.
  12. Spin cells at 15,000× *g* for 30 min at 4 °C. Transfer supernatant to fresh tube. This is the cell extract.
*Note: Perform steps C13–C17 in a cold room.*
  13. Prepare 2 TALON superflow metal affinity columns, one for each enzyme, by packing 3 mL of resin into a Econo-Column chromatography column and equilibrating with 10 mL of wash buffer 1.
  14. Apply the cell extract obtained in step C12 to the column three times. Each time, incubate the column with the extract for 15–20 min on a gentle rocking platform. Save all flowthroughs.
  15. Wash the column with 10 mL of wash buffer 1 three times. Save all washes.
  16. Wash the column with high salt wash buffer to remove nonspecific interactions. Save the wash.
  17. Elute the bound proteins with 3 mL elution buffer in five fractions.
  18. Run 100 μL of each saved wash, flowthrough, and elution on an SDS-PAGE gel and visualize with Coomassie blue.
  19. Combine the relevant fractions (usually 1 and 2, see Figure 1) and buffer exchange with PBS pH 7.4 using a 30 kD Amicon concentrator. Use a total dilution factor of 200 and bring the total volume down to 1 mL. Confirm pH is approximately 7.4 with strips.
  20. Aliquot the combined fractions into PCR tubes (50 μL per tube) and flash freeze in liquid nitrogen. Transfer aliquots to a -80 °C freezer for long-term storage.
    **Figure 1.**
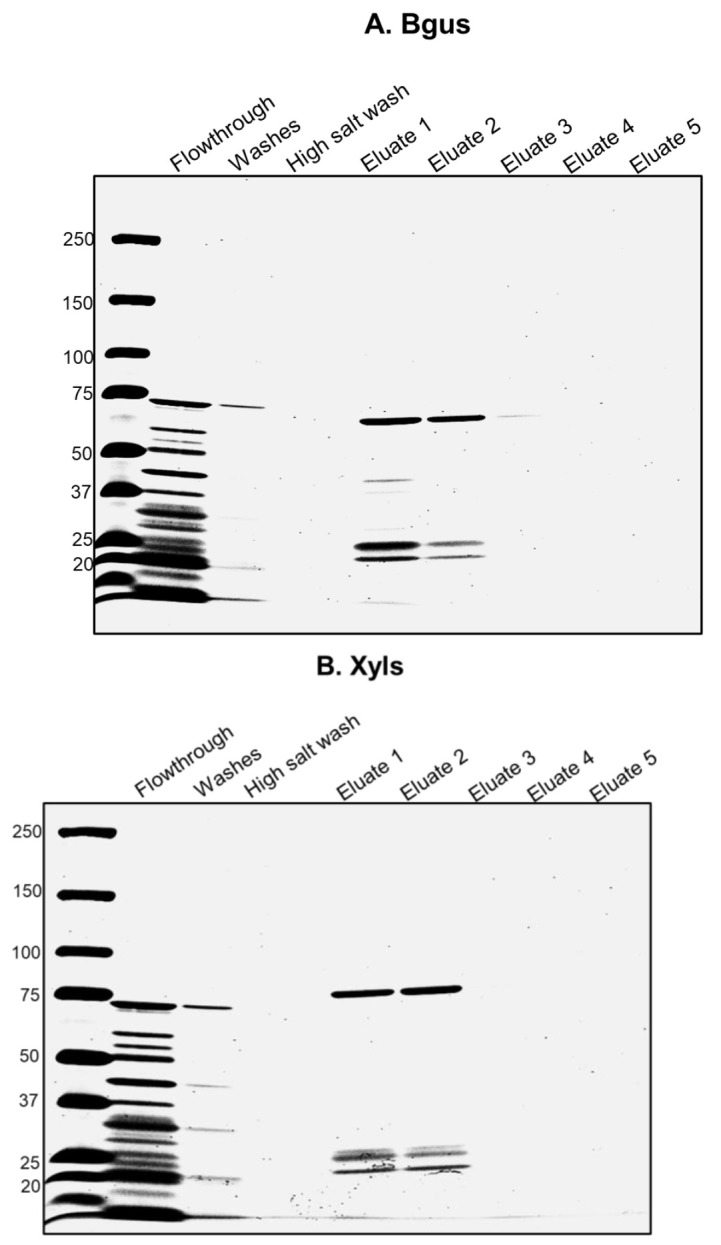
Purification of Bgus and Xyls. A. Column-purified Bgus is shown here in Eluates 1 and 2 and runs at ~66 kD in a 3%–15% gradient SDS-PAGE gel stained with Coomassie blue. B. Column-purified Xyls is shown here in Eluates 1 and 2 and runs at ~80 kD in a 3%–15% gradient SDS-PAGE gel stained with Coomassie blue.
- **Digestion of α-Dystroglycan (α-DG)**
  1. Use wheat germ agglutinin (WGA) agarose to enrich α-DG from animal tissues, as follows:
    - Harvest 1 g of skeletal muscle from C57BL/6J mice. Pour liquid nitrogen into a mortar and pestle and crush the muscle in it.
*Note: Take muscles from the legs after euthanizing mice by cervical dislocation. Briefly, remove the skin by making a small cut in the mid-dorsal or ventral region and peel it off the mouse; then, using scissors and forceps, take all muscles from the legs including quadriceps, tibialis anterior, and soleus, until bone is visible.*
    - Transfer powdered muscle into a vial and allow to warm for 3–5 min, either on ice or in a cold room.
*Note: Vial should be able to withstand cold liquid nitrogen. We use plastic liquid scintillation vials.*
    - Add 10 mL of solubilization buffer and mince the tissue in a polytron homogenizer for 15 s at speed 4. Repeat this three times.
    - Transfer solution to a 50 mL glass homogenizer and macerate tissue by moving the plunger in and out of the solution at least 10 times.
*Note: For macerating tissue with an automated overhead stirrer (instead of doing it by hand), use speed 4.*
    - Transfer homogenized material to a 50 mL Falcon tube and incubate on a rotator for 1 h at 4 °C.
    - Spin the material for 30 min at 20,000× *g* at 4 °C. Transfer supernatant to new tube.
    - Equilibrate 500 μL of WGA beads with 5 mL of solubilization buffer. Spin down beads at 1,500× *g* for 5 min. Remove buffer.
    - Combine material obtained in step D1e with the equilibrated WGA beads and incubate for 16 h on rotator at 4 °C.
    - Spin down beads at 1,500× *g* for 5 min at 4 °C and save the supernatant as WGA-void fraction.
    - Wash WGA beads with 5 mL of WGA wash buffer by incubating for 5 min on a rotator and then spinning beads at 1,000× *g* for 10 min at 4 °C. Repeat this wash step four times.
    - α-DG is now on the WGA beads. Add 1 mL of WGA elution buffer to elute α-DG from 500 μL of WGA beads and incubate at 4 °C for 1 h on a rotator.
    - Spin down beads at 1,500× *g* for 5 min at 4 °C. α-DG is now in the supernatant or eluate.
  2. Buffer exchange 500 μL of the WGA eluate with sodium acetate (pH 5.5) using a 30 kD Amicon concentrator. Use a total dilution factor (DF) of 80 for successfully changing the pH. **Critical:** This step is essential for obtaining the pH (5.5) at which both enzymes work.
*Note: For buffer exchange, follow these steps:*
    - Add 1.5 mL of sodium acetate buffer to 500 μL of WGA eluate in the upper reservoir and spin at 2,000× *g* for 4 min at 4 °C (DF_1_ = 4). Approximately 700 μL remains in the upper reservoir. Discard the solution collected in the centrifuge tube.
    - Add 2.1 mL of sodium acetate buffer to the 700 μL solution from the previous step (DF_2_ = 4). Spin at 2,000× *g* for 4 min at 4 °C. Approximately 500 μL remains in the upper reservoir.
    - Add 2 mL of sodium acetate buffer to the 500 μL solution obtained in the previous step (DF_3_ =5). Spin at 2,000× *g* for 4 min at 4 °C. The solution in the upper reservoir (~ 500 μL) is now at pH 5.5 and the total dilution factor used is 80 (DF_1_ × DF_2_ × DF_3_ = 4 × 4 × 5 = 80)
  3. Add 10 mM β-mercaptoethanol (~ 0.7 μL from 12.8 M stock) to the buffer-exchanged eluate and heat for 5 min at 99 °C. **Critical:** This step is essential to remove disulfide bridges and unfold the protein to make matriglycan accessible for digestion.
  4. Add all protease inhibitors (at final concentrations given in the Recipes) after the solution is cooled down.
  5. Thaw the 50 μL of enzyme aliquots on ice and add them to the above mixture.
  6. Aliquot 100 μL as the initial timepoint (T_o_) and incubate the rest of the mixture at 75 °C for 16 h with shaking at 600 rpm in a thermomixer.
*Note: Aliquots from other timepoints can also be taken (e.g., at 4, 6, and 10 h, for T_4_, T_6_, and T_10_, respectively) to check the rate of progression of the digestion. However, for complete digestion of matriglycan on DG, we recommend incubating the samples for 16–23 h (Figure 2E).*
    **Figure 2.**
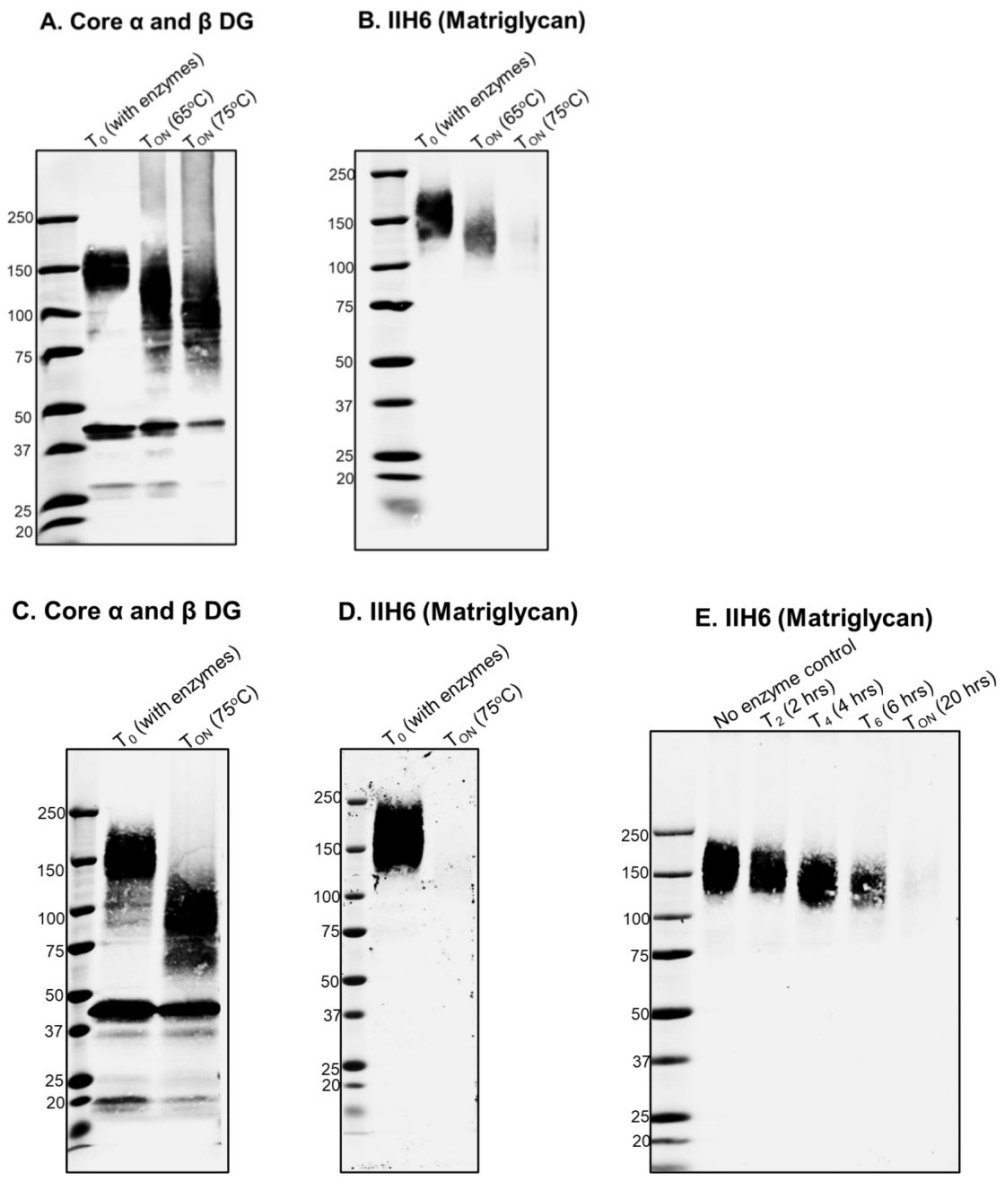
Digestion of matriglycan by Bgus and Xyls. A, B. Wheat germ agglutinin (WGA) eluate from mouse skeletal muscle digested with Bgus and Xyls at 65 °C and 75 °C. T_ON_ indicates overnight digestion of 16 h. The digestion products are immunoblotted with (A) core DG antibody AF6868 1:200 and (B) anti-matriglycan antibody, IIH6, 1:100. C, D. WGA eluate of mouse skeletal muscle digested with Bgus and Xyls at 75 °C overnight (T_ON_, 16 h) and immunoblotted with (C) AF6868 1:200 and (D) IIH6 1:100. E. Progress of digestion over time, as monitored at 2 (T_2_), 4 (T_4_), 6 (T_6_), and 20 h (T_20_); immunoblotting is with antibody against matriglycan IIH6 (1:100).
- **SDS-PAGE and western blotting**
  We analyze the progression and effectiveness of digestion by running the samples in 3%–15% SDS-PAGE gradient gels and performing immunoblotting on PVDF membranes, using antibodies against matriglycan (IIH6, 1:100), against core α-DG (AF6868 1:200) (Figure 2), and/or laminin overlay and solid phase laminin binding assay (Walimbe et al., 2020).

## Data analysis

Upon successful digestion, clear shifts in the molecular weights of α-DG and matriglycan are observed. We also measure changes in laminin binding by performing laminin overlay and solid phase binding analysis (Walimbe et al., 2020, Figure 5D and 5E). Blots are scanned using a Li-Cor Odyssey imaging system and fluorescence detection, and images are analyzed using the Image Studio software. A detailed description of data analysis was provided previously (Briggs et al., 2016, Supplementary figure 1).

## Validation of protocol

We have routinely used this method to identify matriglycan and found it to be robust and reproducible (Okuma et al., 2023; Briggs et al., 2016; Sheikh et al., 2020; Walimbe et al., 2020). Additional data validating our method is provided in Figure 1 and Figure 2.

## General notes and troubleshooting

**General notes**

We have found that these enzymes are most effective when freshly purified.

**Troubleshooting**

If the enzymes are 4–5 months old and do not seem to work, it is best to purify fresh samples.

## Supplementary information

The following supporting information can be downloaded here:
